# RIP3 attenuates the pancreatic damage induced by deletion of ATG7

**DOI:** 10.1038/cddis.2017.313

**Published:** 2017-07-13

**Authors:** Xiaodong Zhou, Li Xie, Leizhou Xia, Frank Bergmann, Markus W Büchler, Guido Kroemer, Thilo Hackert, Franco Fortunato

**Affiliations:** 1 Department of General, Visceral and Transplantation Surgery, University Clinic Heidelberg, Germany; 2 Section Surgical Research, University Clinic Heidelberg, Germany; 3 Affiliated People's Hospital of Jiangsu, University Zhenjiang, Jiangsu, China; 4 Institute of Pathology, University Clinic Heidelberg, Germany; 5 Equipe 11 labellisée par la Ligue contre le Cancer, Centre de Recherche des Cordeliers, Paris, France; 6 INSERM, U1138, Paris, France; 7 Université Paris Descartes, Sorbonne Paris Cité, Paris, France; 8 Université Pierre et Marie Curie, Paris, France; 9 Pôle de Biologie, Hôpital Européen Georges Pompidou, AP-HP, Paris, France; 10 Cell Biology and Metabolomics platforms, Gustave Roussy Cancer Campus, Villejuif, France; 11 Karolinska Institute, Department of Women's and Children's Health, Karolinska University Hospital, Stockholm, Sweden

## Abstract

Invalidation of pancreatic autophagy entails pancreatic atrophy, endocrine and exocrine insufficiency and pancreatitis. The aim of this study was to investigate whether depletion of Rip3, which is involved in necroptotic signaling, may attenuate the pancreatic atrophy and pancreatitis resulting from autophagy inhibition. Autophagy and necroptosis signaling were evaluated in mice lacking expression of Rip3 in all organs and Atg7 in the pancreas. Acinar cell death, inflammation and fibrosis were evaluated by using of a compendium of immunofluorescence methods and immunoblots. Mice deficient for pancreatic Atg7 developed acute pancreatitis, which progressed to chronic pancreatitis. This phenotype reduces autophagy, increase apoptosis and necroptosis, inflammation and fibrosis, as well as premature death of the animals. Knockout of Rip3 exacerbated the apoptotic death of acinar cells, increased tissue damage, reduced macrophage infiltration and further accelerated the death of the mice with Atg7-deficient pancreas. The pancreatic degeneration induced by autophagy inhibition was exacerbated by Rip3 deletion.

Autophagy is an evolutionarily conserved process; it is tightly regulated and activated under cellular stress and energy depletion in order to sequester cytoplasmic components for degradation/recycling and generation of substrates for bioenergetic reactions.^1, 2^ Autophagy plays a cytoprotective role in keeping cellular homeostasis. Defective autophagy can lead to accumulation of waste proteins and damaged organelles (such as uncoupled mitochondria), along with ROS production, DNA damage and NF*κ*B activation, culminating in cell death by apoptosis or necrosis and inflammatory responses.^3^

Inactivation of autophagy can be achieved by deletion of Atg genes, which are essential for this process. Suppression of autophagy in pancreatic cells causes local tissue destruction, leading to a variable degree of insufficiency of the endocrine and exocrine pancreas. Inactivation of floxed Atg5 in the pancreas by means of the p48-Cre recombinase expressed under the control of the *Ptf1a* promoter (Ptf1a-Cre) caused relatively mild pancreatic injury only in male, not in female mice.^4^ In contrast, local inactivation of floxed Atg7 using Pdx1-Cre provoked more severe pancreatic damage with no gender difference. Phenotypic alterations included acinar cell degeneration, pancreatic inflammation and extensive fibrosis, leading to pancreatitis, although no effects on life expectancy were reported.^5^ According to one report, removing floxed Atg7 using Pdx1-Cre in mice expressing a pancreas-specific Kras oncogene improved survival to around 3.6 months and 35% of the remaining mice failed to manifest any PanIN lesions.^6^ Altogether, it appears that depletion of pancreatic Atg7 or Atg5 induces local cell death, thereby triggering pancreatic atrophy and pancreatitis.^4, 7, 8^ Similarly, loss of Atg7 in *β*-cells causes insulin deficiency and type-1 diabetes due to the rarefaction of *β-*cells.^9^

Pancreatic acinar cells are highly efficient in synthesizing and releasing digestive enzymes, meaning that they are continuously exposed to high levels of misfolded or denatured proteins with potentially toxic functions. For this reason, pancreatic acinar cells must quench latent cellular damage by means of autophagy. Disabled autophagy has been linked to multiple distinct pathologies including inflammatory diseases such as pancreatitis.^2, 4, 10^

Chronic pancreatitis (CP) is initiated by acinar cell necrosis driving subsequent pancreatic fibrogenesis, according to the necrosis–fibrosis hypothesis developed by Kloppel *et al.*^11, 12^ Importantly, some forms of necrosis may be regulated and executed by specific proteins.^13^ One version of regulated necrosis, necroptosis, is executed by a kinase, receptor interacting proteins 3 (Rip3), and its downstream phospho-rylation target mixed lineage kinase domain-like protein (Mlkl). Phosphorylated Rip3 can recruit and phosphorylate Mlkl, causing its translocation to the plasma membrane culminating in its permeabilization and cell death.^14, 15, 16^ The role of Rip3 in pancreatitis is controversial. One study reported that Rip3-deficient mice are resistant against experimental acute necrotizing pancreatitis,^13, 17^ while another study using identical mouse strains and pancreatitis models failed to detect any effect of Rip3.^18^ In contrast, Mlkl knockout clearly protected mice from caerulein-induced AP.^19^ As a possibility, Mlkl deletion may be more efficient in attenuating necroptosis-associated disease because Rip3 (and its upstream activator Rip1) have additional functions, for instance in the activation of caspase-8 and NF*κ*B.^16, 18^ This implies that the inactivation of Rip3 and Rip1 would not only inhibit necroptosis (which is a desired goal) but would also stimulate apoptosis and inflammation (which would be an undesired ‘side effect’).

Here, we characterized a novel system for suppressing pancreatic autophagy, by inactivating floxed Atg7 with the Crep48 system. This model was characterized by a particularly rapid destruction of the pancreas with multiple signs of apoptotic and necrotic cell death, AP and CP that caused premature death of the animals. Depletion of Rip3 furthermore exacerbated this phenotype, creating an interesting precedent in which the inhibition of this necroptosis mediator actually aggravates an organ defect caused by autophagy inhibition.

## Results

### Loss of pancreatic Atg7 suppresses pancreatic autophagy

**Figure 1:**
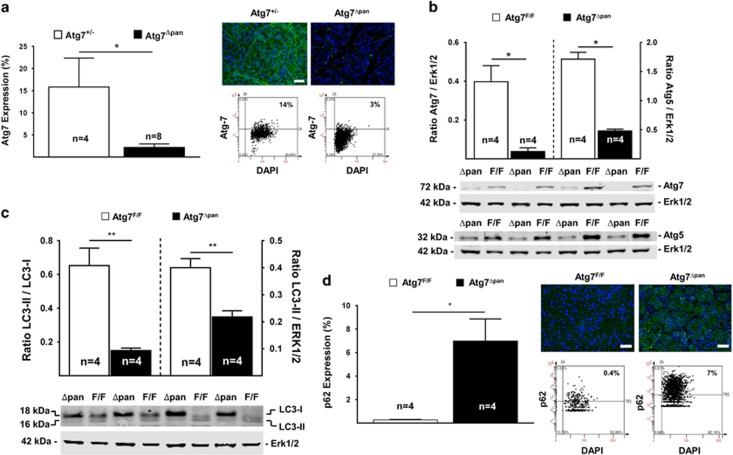
Atg7 depletion reduces autophagy activity. (**a**) Atg7 quantitation and representative IF/scattergram microphotographs of Atg7^F/F^ (*n*=4) and Atg7^Δpan^ (*n*=8) pancreatic tissue stained for DAPI (blue) and Atg7 (green) (anti-Atg7 sc-33211, 1/50, scale bar=50 *μ*m). (**b**) Evaluation of Atg7^F/F^ (*n*=4) and Atg7^Δpan^ (*n*=4) pancreatic Atg7 and Atg5 level by immunoblot analysis using the ratio of Atg7/Atg5 and Erk2 (anti-Atg7 sc-33211, 1/200 dilution, anti-Erk2 sc-154, 1/200). (**c**) Evaluation of Atg7^F/F^ (*n*=4) and Atg7^Δpan^ (*n*=4) of LC3-1 to LC3-II, by WB analysis with either the ratio of LC3-II and LC3-I and LC3-II and Erk2 (anti-LC3 Nanotools 5F10, 1/200). (**d**) p62 quantitation and representative IF/scattergram microphotographs of Atg7^F/F^ (*n*=4) and Atg7^Δpan^ (*n*=4) pancreatic tissue stained for DAPI (blue) and p62 (green)(anti-p62 sc-25575, 1/50, scale bar=50 *μ*m). Data are mean±S.E.M. for the numbers of animals as indicated in the graph, *P<0.05, **P<0.01

We determined whether pancreatic Atg7 deletion is sufficient to trigger CP. For this, *Atg7*-floxed mice (*Atg*7^F/F^) were bred with *Ptf1a/p48-Cre* mice to generate a pancreas-specific Atg7 knockout (*Atg7*^Δpan^),^20, 21^ which was confirmed by PCR of genomic DNA (Supplementary Figure S1A). Immunoblot of homozygous Atg7^Δpan^ mice indicated that Atg7 was present in the liver, yet was undetectable in the pancreas (Supplementary Figure S1B). Deletion of Atg7 from pancreatic cells was determined by quantitative IF for *Atg7*^Δpan^ mice compared with heterozygous *Atg7*^+/−^ mice (Figure 1a). Loss of Atg7 and Atg5 by *Atg7*^Δpan^ were confirmed by immunoblot image (Figure 1b). Pancreata from *Atg7*^Δpan^ mice exhibited a marked reduction in the abundance of LC3-II and hence the LC3-II/LC3-I or LC3-II/Erk1/2 ratios (Figure 1c), as well an accumulation of STQM1/p62, as detected by IF (Figure 1d) and immunoblot (Supplementary Figure S1C). Accumulation of STQM1/p62 in form of cytoplasmic dots has been previously reported following impaired autophagy signaling.^22^ Altogether, our data indicate that the autophagic process was disabled in the pancreas of *Atg7*^Δpan^ mice.

### Loss of pancreatic Atg7 causes CP

**Figure 2:**
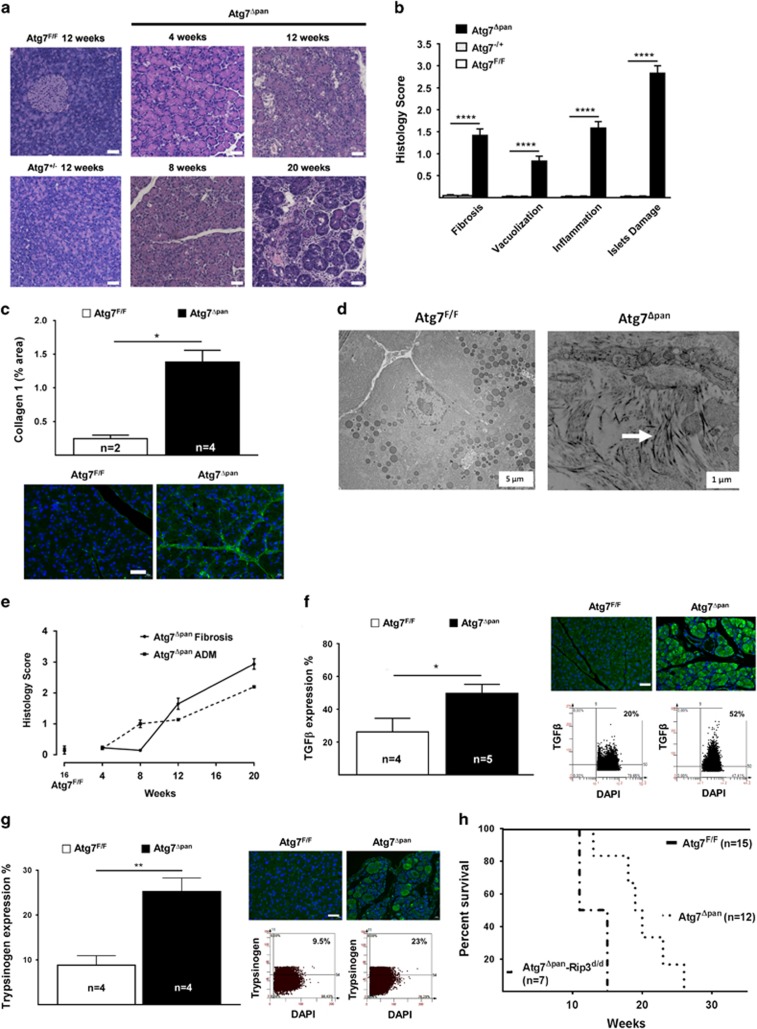
Atg7 depletion induces chronic pancreatitis. (**a**) Representative H&E staining of pancreatic tissue demonstrated that loss of pancreatic Atg7 increased pancreatic tissue damage in Atg7^Δpan^ mice (× 20 objective; scale bar=20 *μ*m). (**b**) Histopathological evaluation of the pancreatic tissue included: For fibrosis: 0, no fibrosis; 1, mild fibrosis; 2, moderate fibrosis; 3, severe fibrosis. For vacuolization: 0, no vacuoles to 3, maximal vacuoles. For inflammation: 0, absent to 3, high infiltration. For Islets damage: 0, no islets damage to 3, severe islets damage, as described previously.^2^ (**c**) Loss of pancreatic Atg7 induces fibrosis. Atg7 quantitation and representative IF microphotographs of Atg7^F/F^ (*n*=2) and Atg7^Δpan^ (*n*=4) pancreatic tissue stained for DAPI (blue) and Collagen-1 (green) (anti-collagen, ab6308, 1/300, scale bar=50 *μ*m). (**d**) Representative transmission electron microscopy image of pancreatic acinar cells from control and Atg7-depleted mice. Arrows indicate collagen fibers indicating tissue fibrosis after loss of pancreatic Atg7. (**e**) Loss of pancreatic Atg7 increased severity of fibrosis and ADM. Histopathology scores were age dependent plotted for fibrosis (continuous line) and ADM (broken line). (**f**) Induction of fibrosis was accompanied with an increased level of the pro-fibrotic factor TGF*β*. TGF*β* quantitation and representative IF/scattergram microphotographs of Atg7^F/F^ (*n*=4) and Atg7^Δpan^ (*n*=5) pancreatic tissue stained for DAPI (blue) and TGF*β* (green) (anti-TGF*β* ab66043, 1/100, scale bar=50 *μ*m). (**g**) Depletion of pancreatic Atg7 increases tissue Trypsinogen/Trypsin expression. Trypsinogen/Trypsin quantitation and representative IF/scattergram microphotographs of Atg7^F/F^ (*n*=4) and Atg7^Δpan^ (*n*=4) pancreatic tissue stained for DAPI (blue) and Trypsinogen/Trypsin (green) (anti-Trypsinogen cs-67388, 1/50, scale bar=50 *μ*m). (**h**) Loss of pancreatic Atg7 reduces survival. Survival rate was analyzed by using Kaplan–Meier analysis comparing survival in Atg7^F/F^ (continuous line), Atg7^Δpan^ (broken line) and Atg7^Δpan^-Rip3^d/d^ (broken/continuous line). Data are mean±S.E.M. for the numbers of animals as indicated in the graph, *P<0.05, **P<0.01, ****P<0.0001

Hematoxylin–eosin staining and histopathology scoring revealed a remarkable increase in acinar cell vacuolization, inflammatory infiltration, fibrosis and islet damage in *Atg7*^Δpan^ mice compared with *Atg7*^F/F^ or heterozygous *Atg7*^*+/d*^ (Figure 2a and 2b). Pancreatic fibrosis is one of the diagnostic criteria for CP.^11^ Collagen detection by IF confirmed an increase in fibrosis in *Atg7*^Δpan^ mice compared with *Atg7*^F/F^ mice (Figure 2c). Collagen fibers were also detectable in *Atg7*^Δpan^ (but not in Atg7^F/F^) pancreata by electron microscopy (EM) (Figure 2d). Fibrosis was not found at 8 weeks of age, but increased substantially after 12 and 20 weeks of age in *Atg7*^Δpan^ mice, supporting the conclusion that AP transforms into CP (Figure 2e). *Atg7*^F/F^ or the heterozygous *Atg7*^+/d^ mice did not show any visible signs of fibrosis. TGF-*β*, which contributes to pancreatic fibrogenesis,^23, 24, 25, 26, 27, 28^ was significantly elevated in 8-week-old Atg7^Δpan^ mice (Figure 2f).

Both the exocrine and endocrine pancreatic tissues underwent severe destruction, explaining the premature mortality of Atg7^Δpan^ mice. The median survival of both male and female *Atg7*^Δpan^ mice was 25 weeks, contrasting with control *Atg7*^F/F^ mice or heterozygous *Atg7*^+/−^ littermates that all lived longer than 50 weeks (Figure 2h). The body weights of both male and female *Atg7*^Δpan^ mice were significantly lower compared with *Atg7*^F/F^ controls (Supplementary Figure S2A). These results are substantially different from other recently reported mouse models of pancreatic autophagy inhibition (Atg5-Crep48, Atg5-CrePdx1 or Atg7-CrePdx1), which manifested a less severe pancreatitis, longer survival and major gender differences.^4, 7^ Pancreatic *α*-amylase was strongly reduced in 12-week-old *Atg7*^Δpan^ mice compared with Atg7^F/F^ controls, indicating a major pancreatic exocrine insufficiency (Supplementary Figure S2B). Similarly, in Atg7^Δpan^ mice, serum *α*-amylase and lipase levels increased significantly after 4 weeks of age, and further decreased after 20 weeks of age, suggesting a transition from AP to CP. In addition, serum glucose and triglyceride levels were markedly increased after 21-week-old *Atg7*^Δpan^ mice, indicating an endocrine insufficiency (Supplementary Figure S2C). Trypsin/Trypsinogen activation, one of the hallmarks of pancreatitis, is highly increased in acinar cells of *Atg7*^Δpan^ mice compared with *Atg7*^F/F^ controls, as detected by IF (Figure 2g) and immunoblot image (Supplementary Figure S2D).

Myeloperoxidase (MPO), a marker for infiltration by neutrophil granulocytes and monocytes, increased as early as at 8 weeks and then remained elevated in Atg7^Δpan^ mice (Supplementary Figure S2E). In contrast, pancreatic macrophage infiltration peaked at 8 weeks, followed by a decrease to normal levels at 20 weeks of age (Supplementary Figure S2F). The formation of acinar-to-ductal cell metaplasia (ADM), a reprogramming process that induces acinar cell trans-differentiation into a ductal-like cell type, may represent the precursors for PanIN lesions.^29, 30^ Similar to fibrosis, ADM were not detectable at 4 weeks of age, yet increased over time in *Atg7*^Δpan^ mice (Figure 2e). Altogether, these histopathological analyses indicate that lack of Atg7 expression in pancreatic acinar cells triggers AP that progressively develops into CP with signs of pre-malignancy.

### Ablation of Atg7 increases acinar cell apoptosis and necroptosis

**Figure 3:**
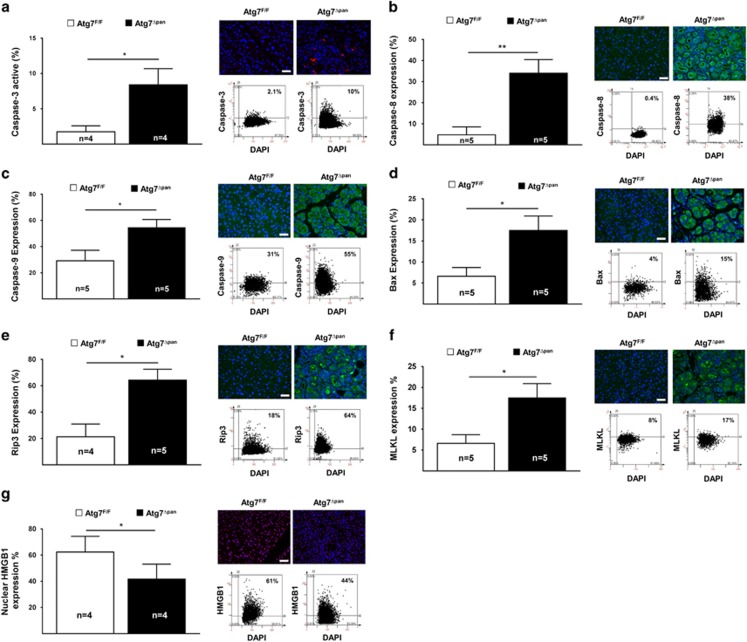
**A**utophagy-deficient mice showed increased activity of apoptosis and necroptosis. (**a**) Depletion of Atg7 increased pancreatic caspase-3 in 20-week-old Atg7^Δpan^ mice. Caspase-3 quantitation and representative IF microphotographs of Atg7^F/F^ (*n*=4) and Atg7^Δpan^ (*n*=4) pancreatic tissue stained for DAPI (blue) and Caspase-3 (red) (anti-active caspase-3 ab2302, 1/50, scale bar=50 *μ*m). (**b**) Reduced pancreatic Atg7 level increased the expression of caspase-8 in 12-week-old Atg7^Δpan^ mice. Caspase-8 quantitation and representative IF microphotographs of Atg7^F/F^ (*n*=5) and Atg7^Δpan^ (*n*=5) pancreatic tissue stained for DAPI (blue) and Caspase-8 (green) (anti-active caspase-8 Novusbio NB100-56116, 1/1000, scale bar=50 *μ*m). (**c**) Increased the expression of caspase-9 in 12-week-old Atg7^Δpan^ mice. Caspase-9 quantitation and representative IF microphotographs of Atg7^F/F^ (*n*=5) and Atg7^Δpan^ (*n*=5) pancreatic tissue stained for DAPI (blue) and Caspase-9 (green) (anti-caspase-9 Novusbio NB100-56118, 1/1000, scale bar=50 *μ*m). (**d**) Reduced pancreatic Atg7 level increased the expression of Bax in 12-week-old Atg7^Δpan^ mice Bax quantitation and representative IF microphotographs of Atg7^F/F^ (*n*=5) and Atg7^Δpan^ (*n*=5) pancreatic tissue stained for DAPI (blue) and Bax (green) (anti-Bax sc-526, 1/50, scale bar=50 *μ*m). (**e**) Reduced pancreatic Atg7 level increased the expression of necroptotic protein Rip3 in 20-week-old Atg7^Δpan^ mice. Rip3 quantitation and representative IF microphotographs of Atg7^F/F^ (*n*=5) and Atg7^Δpan^ (*n*=5) pancreatic tissue stained for DAPI (blue) and Rip3 (green) (anti-Rip3 ab62344, 1/200, scale bar=50 *μ*m). (**f**) Reduced pancreatic Atg7 level increased the expression of necroptotic protein Mlkl in 20-week-old Atg7^Δpan^ mice. Mlkl quantitation and representative IF microphotographs of Atg7^F/F^ (*n*=5) and Atg7^Δpan^ (*n*=5) pancreatic tissue stained for DAPI (blue) and Mlkl (green) (anti-Mlkl ab194699, 1/100, scale bar=50 *μ*m). (**g**) Reduced pancreatic Atg7 level increased the expression of necroptotic protein Hmgb1 in 20-week-old Atg7^Δpan^ mice. Hmgb1 quantitation and representative IF microphotographs of Atg7^F/F^ (*n*=5) and Atg7^Δpan^ (*n*=5) pancreatic tissue stained for DAPI (blue) and Hmgb1 (red) (anti-Hmgb1 ab18256, 1/1000, scale bar=50 *μ*m). Data are mean±S.E.M. for the numbers of animals as indicated in the graph, *P<0.05, **P<0.01

We next investigated whether the ablation of Atg7 in pancreatic acinar cells would induce apoptosis and necroptosis. The expression of the proteolytically mature form of caspase-3 (Figure 3a), as well as the enzymatic activity of caspase-3 (Supplementary Figure S3A), were significantly increased in *Atg7*^Δpan^ compared with *Atg7*^F/F^ pancreata. Very similar results were obtained for caspase-8 (Figure 3b), caspase-9 (Figure 3c) and Bax (Figure 3d), which all were activated in *Atg7*^Δpan^ pancreata, suggesting the ignition of both the intrinsic and extrinsic apoptosis pathways. We additionally confirm the expression of cleaved caspase-8 (Supplementary Figure S3B), cleaved caspase-9 (Supplementary Figure S3C) and Bax (Supplementary Figure S3D) by immunblot experiments.

When compared with Atg7^F/F^ controls, Rip3 protein expression was significantly enhanced in pancreatic tissue from Atg7^Δpan^ mice, as determined by quantitative IF (Figure 3e). Mlkl, which was recently described to interact with Rip3 and to participate in TNF*α*-induced pancreatitis-associated necroptosis,^19^ was also significantly elevated in Atg7^Δpan^ pancreata (Figure 3f). High mobility group protein B1 (Hmgb1), a chromatin-binding protein that is released from the nuclei of necrotic cells, was reduced in Atg7^Δpan^ pancreata (Figure 3g).^2, 31^ We also confirmed the expression of Rip3 (Supplementary Figure S3E), Rip1 (Supplementary Figure S3H), Mlkl (Supplementary Figure S3F) and Hmgb1 (Supplementary Figure S3G) by immunoblot.

Collectively, these results indicate that Atg7 inactivation in pancreatic acinar cells is sufficient to cause substantial cell death by apoptosis and necrosis in the affected organ. Moreover, two necroptosis-relevant proteins, Rip3 and Mlkl, were overexpressed in the context of pancreatitis.

### Pancreatic damage induced by Atg7 deletion is exacerbated by removal of Rip3

**Figure 4:**
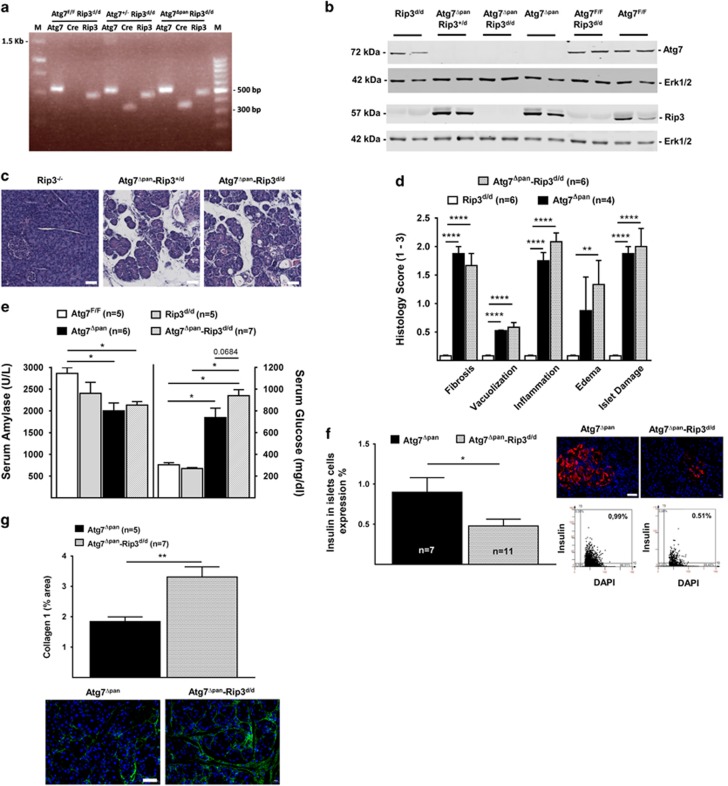
Rip3 deletion did not reverse pancreatic tissue damage by the deletion of pancreatic Atg7. (**a**) Representative PCR agarose gel electrophoreses of mouse tail genotyping. (**b**) Representative immunoblot of double depleted Atg7 and Rip3 in pancreatic tissue of 12-week-old mouse. (**c**) Representative H&E staining of pancreatic tissue demonstrating that loss of pancreatic Atg7 and Rip3. Atg7^Δpan^-Rip3^d/d^ mice (× 20 objective; scale bar=20 *μ*m). (**d**) Histopathology was scored for evidence of injury such as fibrosis, vacuolization, inflammation and islets damage in 12-week-old mice. (**e**) Reduced serum *α*-Amylase and increased serum glucose level in 12-week-old Atg7^Δpan^ and Atg7^Δpan^-Rip3^d/d^ mice. (**f**) Reduced pancreatic insulin level in double-deficient Atg7^Δpan^-Rip3^d/d^ mice compared with Atg7^Δpan^ mice of 12 weeks of age. Insulin quantitation and representative IF microphotographs of Atg7^F/F^ (*n*=7) and Atg7^Δpan^ (*n*=11) pancreatic tissue stained for DAPI (blue) and insulin (red) (anti-insulin B sc-377071, 1/50, scale bar=50 *μ*m). (**g**) Loss of pancreatic autophagy and necroptosis (Atg7^Δpan^-Rip3^d/d^) exacerbate fibrosis compared with Atg7^Δpan^ mice. Atg7 and Rip3 quantitation and representative IF microphotographs of Atg7^Δpan^ (*n*=5) and Atg7^Δpan^-Rip3^d/d^ (*n*=7) pancreatic tissue stained for DAPI (blue) and collagen-1 (green) (anti-collagen ab6308, 1/50, scale bar=50 *μ*m). Data are mean±S.E.M. for the numbers of animals as indicated in the graph, *P<0.05, **P<0.01, ****P<0.0001

Since depletion of pancreatic Atg7 resulted into signs of necrosis and upregulation of Rip3, we investigated whether Rip3 depletion would be able to attenuate pancreatitis. For this, we crossed *Atg7*^Δpan^ with *Rip3*^d/d^ mice. PCR reactions (Figure 4a) and immunoblots of pancreatic extracts from homozygous *Atg7*^Δpan^; *Rip3*^d/d^ mice confirmed that both Atg7 and Rip3 were removed (Figure 4b). Hematoxylin/eosin staining followed by careful scoring of pathological stages failed to reveal any major difference between Atg7^Δpan^-Rip3^d/d^ compared with Atg7^Δpan^ pancreata (Figure 4c). The overall pathological severity score of fibrosis, vacuolization, inflammation, edema and islet damage were similar for *Atg7*^Δpan^-*Rip3*^d/d^, *Atg7*^Δpan^-*Rip3*^+/d^ and *Atg7*^Δpan^ pancreata (Figure 4d). Serum level of *α*-amylase decreased in both *Atg7*^Δpan^ and *Atg7*^Δpan^-Rip3^d/d^ compared with their control mice. Serum glucose, on the other hand, tended to increase in double KO *Atg7*^Δpan^-*Rip3*^d/d^ mice compared with Atg7^Δpan^ animals, although this trend did not reach significance (Figure 4e). However, in the double-deficient Atg7^Δpan^-Rip3^d/d^ mice, pancreatic insulin levels further decreased as compared with Atg7^Δpan^-deficient mice, indicating an aggravation of islet cell dysfunction (Figure 4f). Collagen detection by IF revealed an exacerbated fibrosis in *Atg7*^Δpan^-*Rip3*^d/d^ mice compared with *Atg7*^Δpan^ mice (Figure 4g). The aggravation of the endocrine dysfunction induced by removal of Rip3 might explain the shortened lifespan of *Atg7*^Δpan^-*Rip3*^d/d^ mice compared with *Atg7*^Δpan^ mice (Figure 2h).

### Loss of Atg7 and Rip3 exacerbates apoptosis and alters the immune cell infiltrate

**Figure 5:**
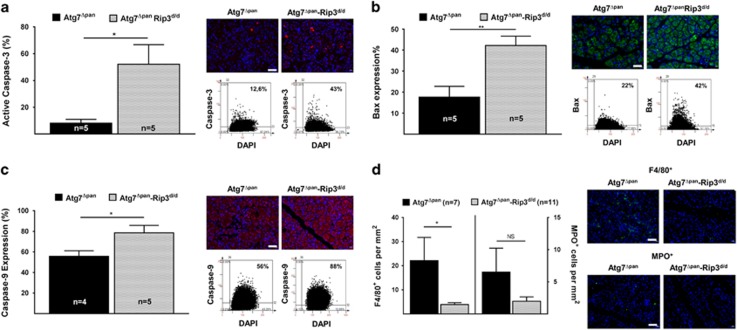
Exacerbated apoptosis and altered inflammation in double-deficient Atg7^Δpan^-Rip3^−/−^ mice. (**a**) Depletion of Rip3 and pancreatic Atg7 (Atg7^Δpan^-Rip3^d/d^) increased pancreatic caspase-3 in 12-week-old mice. Caspase-3 quantitation and representative IF microphotographs of Atg7^Δpan^ (*n*=5) and Atg7^Δpan^-Rip3^d/d^ (*n*=5) pancreatic tissue stained for DAPI (blue) and Caspase-3 (red) (anti-active caspase-3 ab2302, 1/50, scale bar=50 *μ*m). (**b**) Depletion of Rip3 and pancreatic Atg7 (Atg7^Δpan^-Rip3^d/d^) increased pancreatic Bax in 12-week-old mice. Bax quantitation and representative IF microphotographs of Atg7^Δpan^ (*n*=5) and Atg7^Δpan^-Rip3^d/d^ (*n*=5) pancreatic tissue stained for DAPI (blue) and Caspase-3 (red) (anti-Bax (sc-526, 1/50). (**c**) Depletion of Rip3 and pancreatic Atg7 (Atg7^Δpan^-Rip3^d/d^) increased pancreatic caspase-9 in 12-week-old mice. Caspase-9 quantitation and representative IF microphotographs of Atg7^Δpan^ (*n*=5) and Atg7^Δpan^-Rip3^d/d^ (*n*=5) pancreatic tissue stained for DAPI (blue) and Caspase-9 (red) (anti-caspase-9 NB100-56118, 1/1000). (**d**) Reduced infiltration inflammation of macrophages (F4/80, NBP2-12506, 1/75) and early T-lymphocytes (MPO, ab9535, 1/50) in double-deficient Atg7^Δpan^-Rip3^d/d^ (*n*=10) mice compared with pancreatic Atg7^Δpan^ (*n*=7). Data are mean±S.E.M. for the numbers of animals as indicated in the graph, *P<0.05, **P<0.01, NS = no significance

The active form of caspase-3 was significantly increased in double-deficient *Atg7*^Δpan^-*Rip3*^d/d^ compared with *Atg7*^Δpan^ pancreata (Figure 5a). Very similar results were obtained for Bax (Figure 5b) and caspase-9 (Figure 5c), while the active form of caspase-8 exhibited a tendency to increase (Supplementary Figure S3B), indicating accelerated intrinsic and extrinsic apoptosis activity. Nuclear Hmgb1 staining in pancreatic cells was not different between *Atg7*^Δpan^ and *Atg7*^Δpan^-Rip3^d/d^ mice (Supplementary Figure S3C). Surprisingly, macrophage infiltration was significantly reduced in the double-deficient *Atg7*^Δpan^-*Rip3*^d/d^ compared with *Atg7*^Δpan^ pancreata (Figure 5d). Similarly, removal of both Atg7 and Rip3 led to a nonsignificant reduction in MPO positivity with respect to *Atg7*^Δpan^ pancreata (Figure 5d). In addition, T and B lymphocyte infiltration that may be influenced by Rip3-depleted immune cell reprogramming^32^ did not change in the double-deficient *Atg7*^Δpan^-*Rip3*^d/d^ compared with *Atg7*^Δpan^ pancreata (Supplementary Figure 3I). Hence, in contrast to our expectations, Rip3 removal did not reduce pancreatic tissue damage, but rather enhanced this damage, though without an increase in local inflammation and even a reduction in macrophage counts.

## Discussion

The present study reveals that pancreatic Atg7 deletion causes gender-independent severe pancreatitis with progressive cell death, fibrosis and inflammation. *Atg7*^Δpan^ succumbed to the ever-aggravating exocrine and endocrine dysfunction within a few months. Additional removal of Rip3 further accelerated pancreatic tissue damage and the associated premature mortality. Atg7 deletion alone increased necroptosis, apoptosis and inflammation, and additional Rip3 depletion exacerbated pancreatic apoptosis and reduced local macrophage infiltration, in line with the function of Rip3 not only in necroptosis but also in inflammatory responses.^16^

The observation that Atg7-deleted pancreatic acinar cells progressively degenerate and die, as local tissue damage entails acute and chronic inflammation, emphasizes the importance of basal autophagy in maintaining pancreatic homeostasis. Deletion Atg7 in pancreatic acinar cells was sufficient to induce strong acinar cell death associated with AP that later progressed to CP. Such mice manifested local inactivation of the autophagy machinery tied to accelerate cell death by necroptosis and apoptosis, consequent pancreatic atrophy, inflammation and fibrosis, all of which have been recently described, though with a less severe phenotype, in other knockout models.^2, 4, 5, 11, 33^

Atg5 and Atg7 are both essential for autophagy, and lack of either of them close-to-entirely suppresses the autophagic processes. As compared with our results obtained by deletion of Atg7, deletion of pancreatic Atg5 reportedly caused much less severe pancreatic damage with gender-specific pathogenic effects that were more pronounced in males and hardly detectable in females.^4^ Although the gender-specific difference in Atg5-deleted mice remain elusive, the function of Atg7 and Atg5 may independent of autophagy processes.^34^ Thus it is possible that the impaired autophagy-independent function of Atg7 and Atg5 provide additional pancreatic injury and exacerbate cell death signals. The loss of autophagy-independent function may explain the reported differences of Atg7 and Atg5.

Yet another study in which floxed *Atg7* was inactivated by *Cre* expressed under the control of the Pdx1 promotor (another essential pancreatic embryonic transcription factor frequently used for targeting genes to the pancreas), exhibited endocrine and exocrine damage, though again with less severe damage and only 60% of premature mortality.^7^ This is sharp contrast with our findings revealing full (100%) penetrance of the premature mortality phenotype. The exact mechanisms underlying these discrepancies remain elusive. As a possibility, the timing of the Atg7 depletion driven by the two developmental transcription factors p48 and Pdx1 may be important. During pancreatic development, Pdx1 is expressed earlier than p48, and co-expression of these two transcription factors in multipotent progenitor cells is critical for the cellular differentiation of all three main pancreatic cell types (acinar, endocrine and ductal cells).^35^ Using either of them as a driver for Cre, recombination is initiated in both endocrine and exocrine cells of the pancreas. After birth, Pdx1 is progressively restricted to endocrine pancreas, while p48 expression is confined to acinar cells.^36^ Moreover, p48 is more specific for the pancreas than Pdx1, which is also expressed in the duodenum, antral stomach and bile duct.^35^ In the Atg7-p48-Cre or Atg7-Pdx1-Cre mouse models, deletion of Atg7 of pancreas is almost completed before birth, but due to different temporal and spatial expression patterns of p48 and Pdx1, the effectiveness of the Atg7 knockout may be slightly different, hence affecting the severity of pancreatitis and the probability of early death.

Rip1 and Rip3 and activated Mlkl can promote necroptotic cell death in the absence of caspase-8 in multiple disease models.^18^ The regulation of necroptosis is complex and blockage of Rip1 or Rip3 suppresses not only necroptosis but also apoptosis and NF*κ*B-dependent inflammation in a context-dependent fashion. However, neither Rip1 nor Rip3 is required for apoptosis execution.^18^ As mentioned in the Introduction, the contribution of Rip3 to caerulein-induced pancreatitis is highly controversial as it is in other disease models.^13, 17, 18, 37^ In Atg7^Δpan^ mice, removal of Rip3 accelerated signs of pancreatitis, diabetes and reduced mortality rates. Surprisingly, depletion of Rip3 exacerbated pancreatic apoptosis accompanied with reduced macrophage infiltration. In a recent study, loss of Rip3 has been shown to promote tumorigenic T-cell infiltration in the context of Kras-driven pancreatic oncogenesis.^32^ This study showed that loss of Rip3 resulted into decreased tissue infiltration by macrophages but increased T and B lymphocyte infiltration.^32^ Here, Atg7^Δpan^-Rip3^d/d^ mice exhibited reduced pancreatic macrophage infiltration and MPO but no alteration in T and B lymphocyte densities within the pancreatic tissue. The reasons for these discrepancies appear elusive, although the context of the inflammatory responses (cancer *versus* pancreatitis) may be determinant.

In summary, Atg7^Δpan^ and Atg7^Δpan^-Rip3^d/d^ mice were born healthy, and no pancreatic injury was observed up to 4 weeks of age, followed by minor pancreatic damage at 8 weeks of age. It is only at the age of 8–20 weeks that Atg7^Δpan^ mice exhibited strong destruction of their exocrine and endocrine pancreata, thereby compromising the survival of the animals all of which were dead at the age of 24 weeks. Local inactivation of the autophagic machinery then compromised the survival of pancreatic exocrine acinar cells that activated a diverse array of cell death mechanisms including apoptotic and necroptotic pathways. This process was followed by invasion of the pancreas by inflammatory leukocytes from the myeloid lineage, production of the pro-fibrotic factor TGF-*β* and fibrotic degeneration of the tissue, resulting in insufficiency of the exocrine and/or endocrine function of the pancreas and premature death. In this scenario removal of Rip3 (which should block necroptosis) exacerbated apoptosis and fibrosis as it reduced macrophage infiltration. Hence, we conclude that apoptotic cell death mechanisms play a predominant role in our experimental setting.

## Materials and Methods

### Materials

Antibodies were selected according to proven functionality for formalin-fixed paraffin-embedded (FFPE) tissue sections and WB by the seller or by publication records. The following antibodies were used for WB: Erk2 (sc-154), Atg7 (sc-33211), p62 (sc-25575) and Bax (sc-526), all were purchased from Santa Cruz Biotechnology (Heidelberg, Germany). Mouse Rip3 (ab62344) and MLKL (ab194699) were purchased from Abcam (Cambridge, UK). LC3 (5F10, 0231-100) was purchased from Nanotools (Teningen, Germany). Atg5 (AP1812a) was purchased from ABGENT (San Diego, CA, USA).

For IF we used the following antibodies: Atg7 (sc-33211), p62 (sc-25575), Insulin (sc-377071), Bax (sc-526), CD19 (sc-69733) and *α*-Amylase (sc-31869) all were purchased from Santa Cruz Biotechnology. Active caspase-3 (ab2302), Collagen-1 (ab6308), TGF-*β* (ab66043), MPO (ab9535), Rip3 (ab62344), MLKL (ab194699), HMGB1 (ab18256), CD3 (ab5690) and Macrophage Marker (sc-66204) were purchased from Abcam. F4/80 (NBP2-12506), Active/cleaved Caspase-8 (NB100-56116), active/cleaved Caspase-9 (NB100-56118) was obtained from Novus Biologicals (Cambridge, UK). Cleaved Caspase-3 (cs-9661) was purchased from Cell Signaling Technology (Danvers, MA, USA). ATG5 antibody used in IF was same as WB listed above. Secondary anti-rabbit Cy3- or Cy5-conjugated and anti-mouse Cy3- or Cy5-conjugated antibodies were purchased from Medac GmbH (Wedel, Germany) and applied for IF.

For WB secondary goat anti-rabbit IgG-HRP (sc-2054), goat anti-mouse IgG-HRP (sc-2055) and donkey anti-goat IgG-HRP (sc-2020) were purchased from Santa Cruz Biotechnology. Additionally IRDye 680RD Goat anti-mouse (926-68070) and IRDye 800CW goat anti-rabbit (926-32211) obtained from LI-COR (Bad Homburg, Germany) were used for IB. All other chemicals were from Sigma-Aldrich (Deisenhofen, Germany), if not stated otherwise.

### Animals

Atg7-floxed (Atg7^F/F^) mice, kindly provided by H Rossiter, L Eckhart and M Komatsu, were bred with Ptf1a/p48-Cre mice to generate pancreas-specific Atg7^Δpan^ mice that have been similar described previously using the Atg7 or Atg5-Pdx1-Cre model.^7, 8^ Global Rip3 KO (Rip3^d/d^) mice were kindly provided from M Pasparakis and V Dixit (Genentech, South San Francisco, CA).^38, 39^

### Experimental design

Mice were bred under standard conditions and maintained on a C57LB/6 background. Animal breeding was approved by the Institutional Animal Care and Use Committee in accordance with the guidelines of the University of Heidelberg and the Federal Animal Care and Use Board, Karlsruhe, Germany. All mice were sedated with pentobarbital (Nembutal, 60 mg/kg body weight, i.p.) and the whole pancreas was resected, described previously.^2, 31, 40^ Parts of the pancreas were immersed in liquid nitrogen and stored at −80 °C or immediately fixed in 4% buffered formalin solution, as described earlier.^2^ Serum samples were also taken and stored at −80 °C. Serum parameters including *α*-amylase and lipase were determined by our institutional blood analysis center.

### Pancreatic histopathology

FFPE pancreatic tissue were cut into 4-*μ*m-thick sections and stained with hematoxylin and eosin. Histopathological evaluation of the pancreatic tissue included the severity of fibrosis, the severity of inflammation, the activity of inflammation, and the severity and activity of the perineural inflammation as described recently.^41^ Briefly, histopathological evaluation of the pancreatic tissue included: For fibrosis: 0, no fibrosis; 1, mild fibrosis; 2, moderate fibrosis; 3, severe fibrosis. For vacuolization: 0, no vacuoles to 3, maximal vacuoles. For inflammation: 0, absent to 3, high infiltration. For islets damage: 0, no islets damage to 3, severe islets damage, as described previously.^2^

### Genotyping

DNA extraction was accomplished according to the protocol of the Fast Tissue-to-PCR Kit, using mouse tails. Four microliters DNA extracts were added to a 25 *μ*l PCR reaction system containing Dream Taq PCR Master Mix primers and nuclease-free water (Thermo Fisher Scientific, Waltham, MA, USA). DNA amplification was conducted in a Mastercycler personal PCR machine (Eppendorf, Germany), followed by separation on a 2% agarose gel containing DNA Stain G (SERVA, Heidelberg, Germany) under standard DNA electrophoresis conditions and UV illuminator visibility. The primers used in the PCR reaction are listed: Atg7 forward primer: 5′-TGGCTGCTACTTCTG-CAATGATGT-3′, reverse primer: 5′-CAGGACAGAGACCATCAGCTCCAC-3′ p48-cre forward primer: 5′-ACCGTCAGTACGTGAGATATCTT-3′, reverse primer: 5′-ACCTGAAGA-TGTTCGCGATTATCT-3′ Rip3 primer-1: 5′-CGCTTTAGAAGCCTTCAGGTTGAC-3′, Rip3 primer-2: 5′-GCCTGCCCATCAGCAACTC-3′, Rip3 primer-3: 5′-CCAGAGGCCACTTGT-GTAGCG-3′.

### Immunofluorescence

IF was conducted using 4 *μ*m thin FFPE pancreatic tissue sections obtained from Atg7 mice and human specimens and processed as described in detail previously.^2, 31, 40^ All images were processed using the StrataQuest software (TissueGnostics, Vienna, Austria), allowing the quantitation of the total cell numbers from DAPI-positive cells as well as quantitation of target positive cells. In FACS-like scattergrams, every point represents one single cells were plotted according to their Cy3 and Cy5 IF intensity *versus* their DAPI-intensity from the entire tissue. IF-positive cells were gated in the scattergrams according to negative controls (no primary antibody), and the fluorescence intensity was expressed as a percentage of the mean intensity of the DAPI staining and mean intensity of the target protein staining in a FACS-like scattergram approach, as described previously.^2, 31, 40^ Infiltrated macrophages or MPO-positive monocytes were determined by the numbers of positive cell per mm^2^ tissue.

### Immunoblot analysis

Immunoblot analysis was performed in order to evaluate variations in the expression of specific proteins involved in apoptosis, autophagy and necrosis signaling. Human frozen tissues were homogenized on ice as described previously.^2^ Protein loading control was performed with Erk2 (after using a Restore Western Blot Stripping Buffer Plus (Pierce Biotech., Rockford, IL, USA) to ensure equal protein loading, according to the instructions. Protein fragments were processed and analyzed by using computer-assisted software ImageJ (NIH, Bethesda, MD, USA).

### EM observation

Freshly isolated pancreatic tissue pieces were fixed in 3% glutaraldehyde and embedded in Epon. Ultra-thin sections were prepared for microscopy according to a routine procedure described previously.^2^

### Caspase-3 activity

The activity of caspase-3 was measured in pancreatic tissue extracts using a modified assay for measuring caspase activity as described previously.^42^ Results were expressed as the caspase-3 substrate cleavage in release fluorescence units, after subtraction of the nonspecific product formation (substrate plus inhibitor).

### Statistical analysis

Statistical analysis was performed using Student’s *t-*test for each group. For all column data sets, analysis of identified outliers was performed and results were considered significant when *P-*value ≤0.05, indicated with *, using GraphPad Prism 6 software for statistical calculations. (*n*=*x*) represent the numbers of animals *x*, included in this study. All results were reported as mean±S.E.M. (standard error of the mean) as indicated with the significance score (*P<0.05; **P<0.01; ***P<0.001, ****P<0.0001) in the figure legend.
