# Supplementary material for: RIP3 attenuates the pancreatic damage induced by deletion of ATG7
Source: Cell Death Dis. 2017 Jul 13;8(7):e2918–. doi: 10.1038/cddis.2017.313 (PMC5550860; doi:10.1038/cddis.2017.313)
Supplement: Supplementary Figure Legends [file cddis2017313x1.docx]

**Supplemental Figure Legend**

**Supplemental Figure 1: A)** Representative PCR agarose gel electrophoreses of mouse tail genotyping. **B)** Representative immunoblot of depleted Atg7 in pancreatic and liver tissue of 12-weeks-old Atg7^∆pan^ mouse. **C)** Representative pancreatic p62 expression was determined by Immunoblot analysis using the ratio of p62 and ERK1/2, as indicated by the representative SDS-PAGE autoradiograph. Ratios were plotted as means ± SEM for the numbers of animals indicated in the graph; *) p<0.05.

**Supplemental Figure 2: A)** Reduced body weight in pancreatic Atg7 depleted 12-weeks-old Atg7^∆pan^ mouse. **B)** Representative pancreatic α-Amylase expression determined by FACS-like IF quantitation, stained for DAPI (blue) and α-Amylase (red). Representative shown IF images and scattergrams (20x objective; Scale bar = 50 µm). α-Amylase expression values were plotted as means ± SEM for the numbers of patients indicated in the graphs. **C)** Reduced serum α-Amylase and Lipase and increased serum Glucose and Triglyceride in 12-weeks-old Atg7^∆pan^ mouse. **D)** Increased Trypsin activation in pancreatic Atg7 depleted 12-weeks-old Atg7^∆pan^ mouse. Trypsin was determined by Immunoblot analysis using the ratio of Trypsin and ERK1/2, as indicated by the representative SDS-PAGE autoradiograph. Ratios were plotted as means ± SEM for the numbers of animals indicated in the graphs; *) p<0.01. **E)** Loss of pancreatic Atg7 increased inflammation determined by FACS-like IF quantitation over time stained for DAPI (blue) and MPO (green) for 2 to 3 animals per age. MPO-positive cells were blotted per mm^2^ tissue area for Atg7^∆pan^ mice (continues line) and Atg7^F/F^ mice (dashed line), and both lines were determined to be significantly different. Representative shown IF images (20x objective; Scale bar = 50 µm). **F)** Loss of pancreatic Atg7 increased inflammation determined by FACS-like IF quantitation over time stained for DAPI (blue) and macrophage F4/80 (green) for 2 to 3 animals per age. Macrophage-positive cells were blotted per mm^2^ tissue area for Atg7^∆pan^ mice (continues line) and Atg7^F/F^ mice (dashed line), and both lines were determined to be significantly different. Representative shown IF images and scattergrams (20x objective; Scale bar = 50 µm). All results were expressed as means ± SEM, *) *p*≤0.05; **) *p*≤0.01; ***) *p*≤0.001.

**Supplemental Figure 3: A)** Increased Caspase-3 activity in pancreatic Atg7 depleted tissue extract of 12-weeks-old Atg7^∆pan^ mouse. Caspase-3 activity was determined by release of flourescence over time as described previously. **B)** Increased active Caspase-8 of pancreatic tissue extract of Atg7^∆pan^-Rip3^d/d^ 12-weeks-old mouse. Active caspase-8 was determined by immunoblot (anti-caspase-8 Novusbio NB100-56116, 1/1000). as described for caspase-3 using caspase-8 specific substrate. **C)** Increased active Caspase-9 of pancreatic tissue extract of Atg7^∆pan^-Rip3^d/d^ 12-weeks-old mouse. Active caspase-9 was determined by immunoblot (anti-caspase-9 Novusbio NB100-561186, 1/1000). **D)** Increased Bax of pancreatic tissue extract of Atg7^∆pan^-Rip3^d/d^ 12-weeks-old mouse. Bax was determined by immunoblot (anti-Bax sc-526, 1/200). **E)** Increased Rip3 of pancreatic tissue extract of Atg7^∆pan^-Rip3^d/d^ 12-weeks-old mouse. Rip3 was determined by immunoblot (anti-Rip3 ab62344, 1/1000). **F)** Increased Mlkl of pancreatic tissue extract of Atg7^∆pan^-Rip3^d/d^ 12-weeks-old mouse. Mlkl was determined by immunoblot (anti-Mlkl ab194699, 1/500). G**)** Decreased Hmgb1 of pancreatic tissue extract of Atg7^∆pan^-Rip3^d/d^ 12-weeks-old mouse. Hmgb1 was determined by immunoblot (anti- Hmgb1 ab182569, 1/100). **H)** Increased Rip1 of pancreatic tissue extract of Atg7^∆pan^-Rip3^d/d^ 12-weeks-old mouse. Rip1 was determined by immunoblot (anti-Rip1 Novusbio NBP1-77077, 1/500). **I)** T-cells and B-cells content in pancreatic tissue in Atg7^∆pan^ and Atg7^∆pan^-Rip3^d/d^ depleted 12-weeks-old mouse. CD3 positive T-cells and CD19 positive B-cells pancreatic content were determined by FACS-like IF quantitation, stained for DAPI and T-, and B-cells (data not shown). T-, and B-cells tissue content were plotted as means ± SEM for the numbers of patients indicated in the graphs.
