# Supplementary figures and images for: RIP3 attenuates the pancreatic damage induced by deletion of ATG7
Source: Cell Death Dis. 2017 Jul 13;8(7):e2918–. doi: 10.1038/cddis.2017.313 (PMC5550860; doi:10.1038/cddis.2017.313)

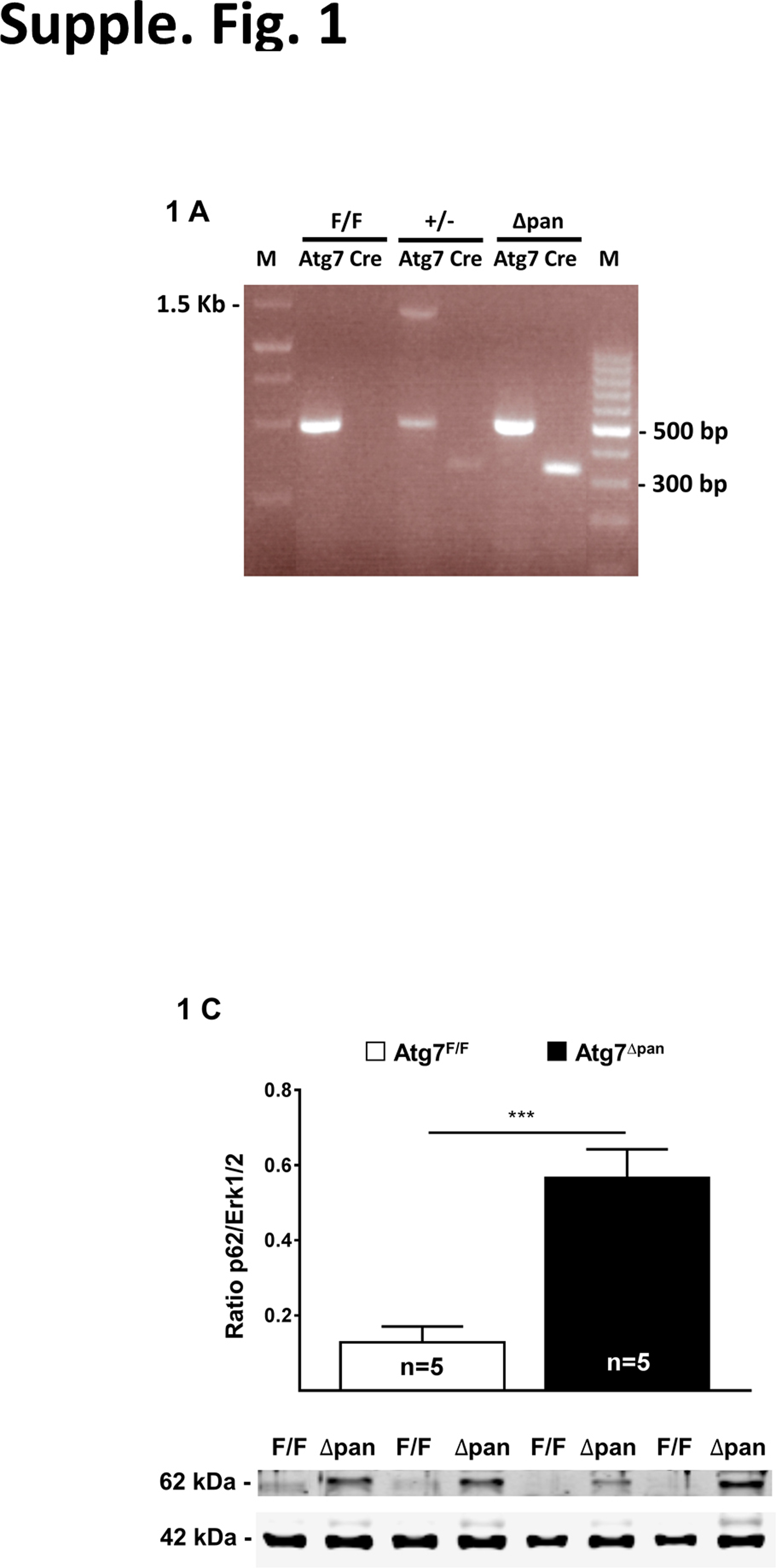

Supplement: Supplementary Figure 1 [file cddis2017313x2.tif]

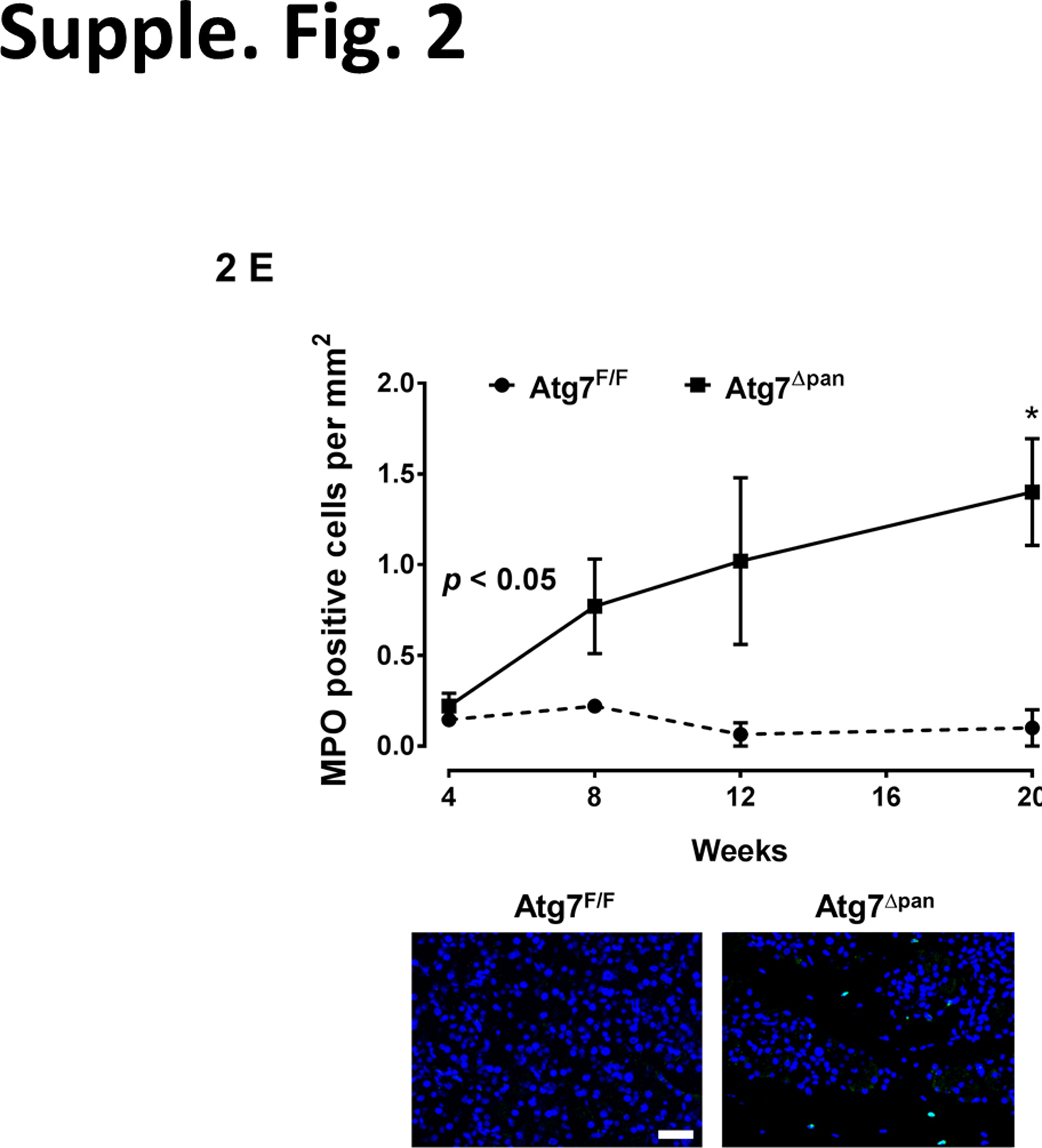

Supplement: Supplementary Figure 3 [file cddis2017313x4.tif]

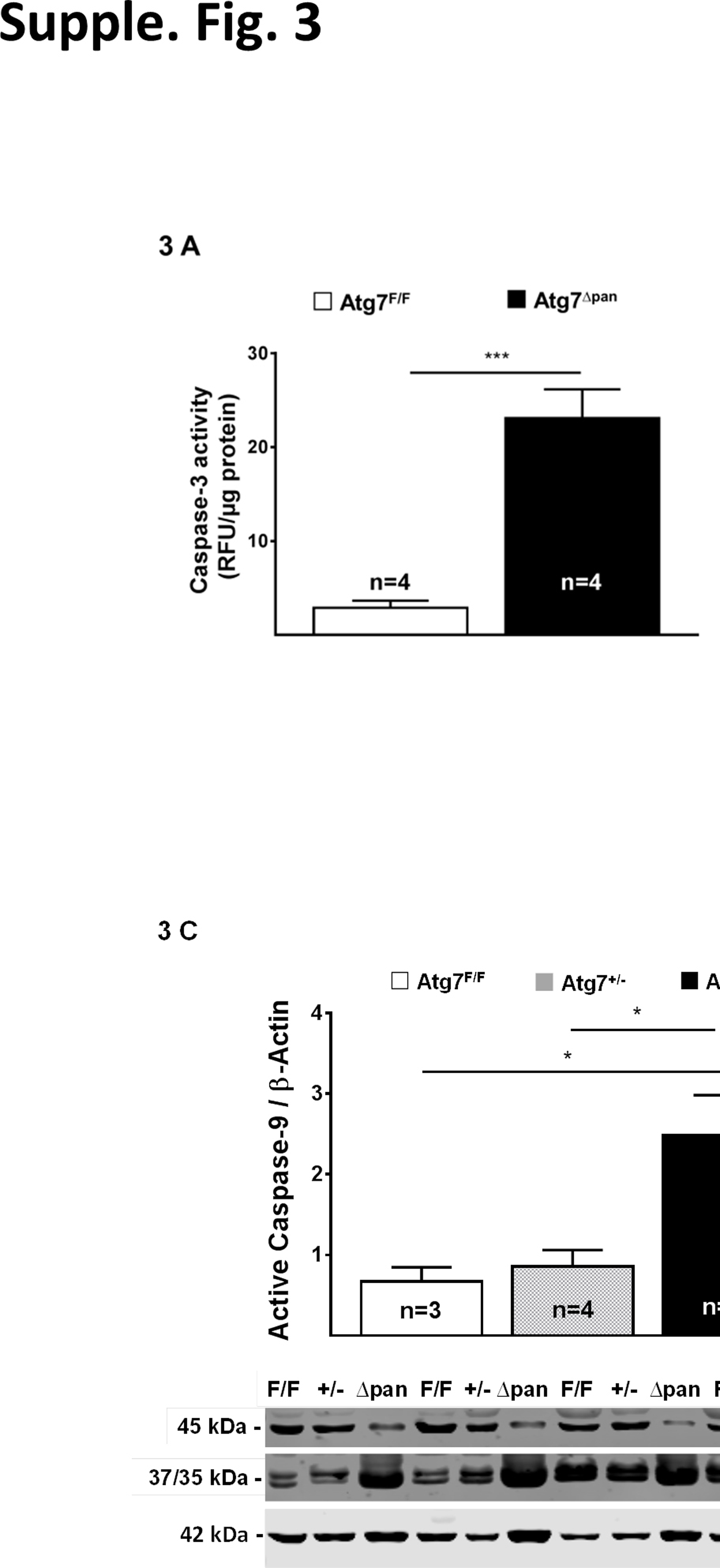

Supplement: Supplementary Figure 4 [file cddis2017313x5.tif]

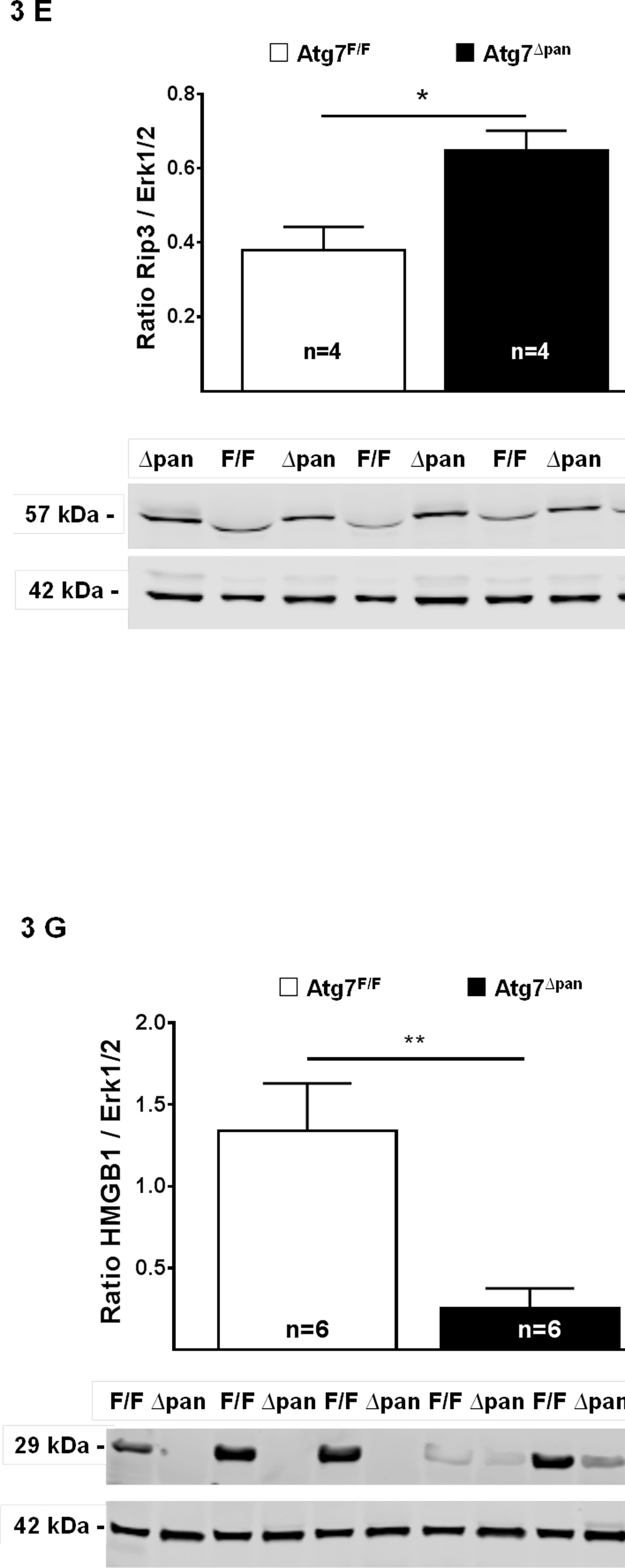

Supplement: Supplementary Figure 5 [file cddis2017313x6.tif]

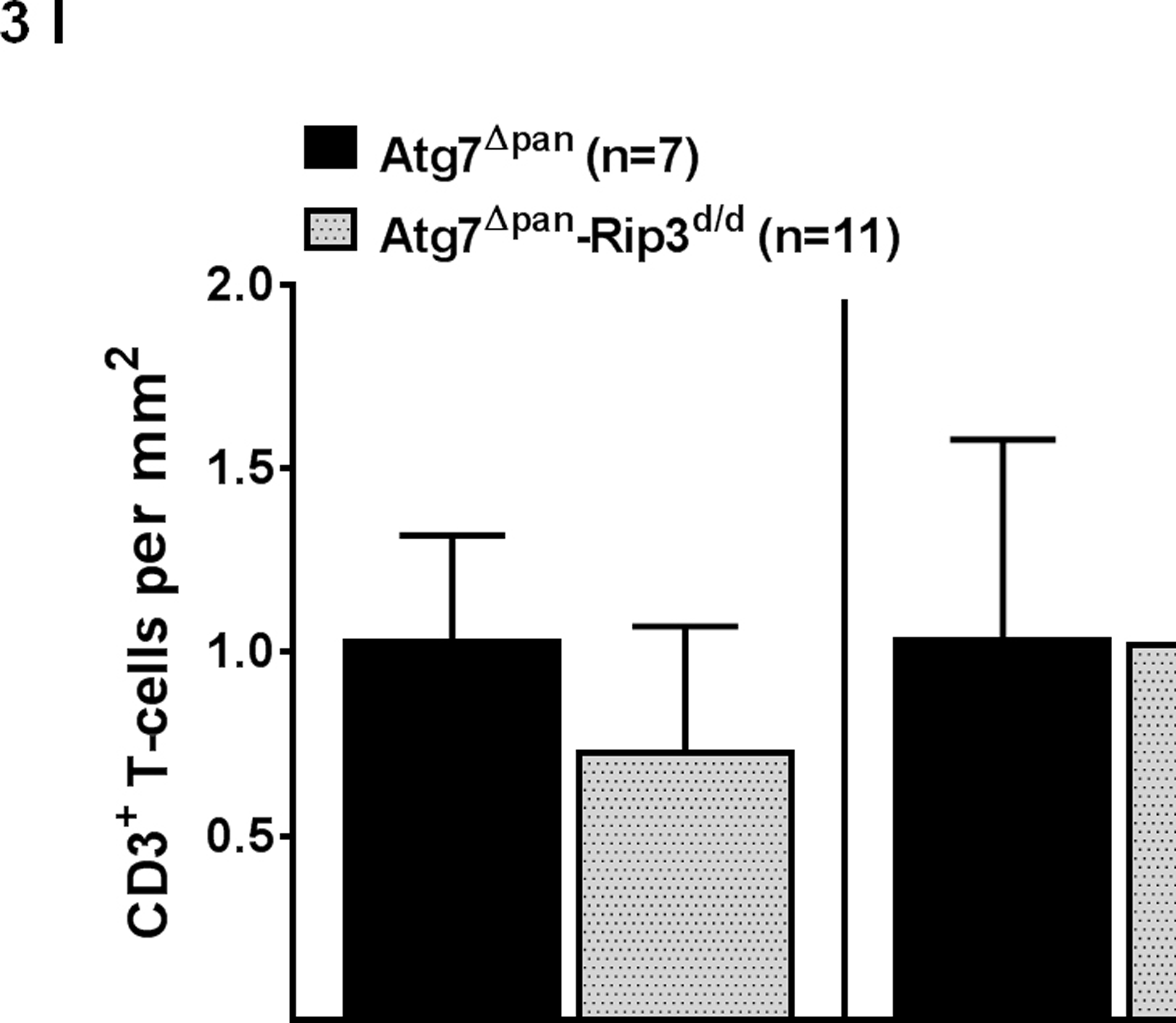

Supplement: Supplementary Figure 6 [file cddis2017313x7.tif]
